# Multi-cohort cross-omics analysis reveals disease mechanisms and therapeutic targets in HTLV-1-associated myelopathy, a neglected retroviral neuroinflammatory disorder

**DOI:** 10.21203/rs.3.rs-5960764/v1

**Published:** 2025-04-29

**Authors:** Johan Van Weyenbergh, Tatiane Assone, Isaac Racine, Soraya Menezes, Fernanda Gonçalves, Víctor Folgosi, Rosa Marcusso, Michel Haziot, Jerusa Smid, Flavia Dahy, Maria Gascon, Arthur Paiva, Bernardo Galvao-Castro, Thessika Araújo, Maria Grassi, Maísa Sousa, Marzia Puccioni-Sohler, Youko Nukui, Simone Kashima, Tim Dierckx, Jean-Claude Twizere, Edward Murphy, Roberta Bruhn, Christophe Pannecouque, Sandra Claes, Evelien Vanderlinden, Dominique Schols, Jurgen Vercauteren, Carolina Alvarez, Giovanni Lopez, Michael Talledo, Eduardo Gotuzzo, Augusto Oliveira, Isabelle Cleynen, Jorge Casseb

**Affiliations:** KU Leuven; Faculdade de Medicina/USP; Laboratory for Complex Genetics, Department of Human Genetics/KU Leuven; Laboratory of Clinical and Epidemiological Virology, Department of Microbiology, Immunology and Transplantation, Rega Institute for Medical Research, KU Leuven; Faculdade de Medicina / Universidade de Sao Paulo; University of São Paulo Medical School - USP; Institute of Infectious Diseases “Emilio Ribas” / Secretaria de Saúde do Estado de São Paulo; Institute of Infectious Diseases “Emilio Ribas” / Secretaria de Saúde do Estado de São Paulo; Institute of Infectious Diseases “Emilio Ribas” / Secretaria de Saúde do Estado de São Paulo; Institute of Infectious Diseases “Emilio Ribas” / Secretaria de Saúde do Estado de São Paulo; Universidade São Judas Tadeu; Hospital Universitário Prof. Alberto Antunes / Universidade Federal de Alagoas; Oswaldo Cruz Foundation; Centro HTLV/Escola Bahiana de Medicina e Saúde Pública; Instituto Gonçalo Moniz/ Fundação Oswaldo Cruz (Fiocruz); Núcleo de Medicina Tropical/ Universidade Federal do Pará; Escola de Medicina e Cirurgia/UNIRIO; Pos-graduation in infecctious diseases/ UFRJ; Hospital das Clínicas da FMUSP/ Universidade de Sao Paulo; University of São Paulo, Ribeirão Preto Medical School, Blood Center of Ribeirão Preto, Ribeirão Preto, SP, Brazil; Laboratory of Clinical and Epidemiological Virology, Department of Microbiology, Immunology and Transplantation, Rega Institute for Medical Research; University of Liege; Vitalant Research Institute; Vitalant Research Institute; Laboratory of Virology and Chemotherapy, Department of Microbiology, Immunology and Transplantation, Rega Institute for Medical Research, KU Leuven; Laboratory of Virology and Chemotherapy, Department of Microbiology, Immunology and Transplantation, Rega Institute for Medical Research, KU Leuven; Laboratory of Virology and Chemotherapy, Department of Microbiology, Immunology and Transplantation, Rega Institute for Medical Research, KU Leuven; University of Leuven; Laboratory of Clinical and Epidemiological Virology, Department of Microbiology, Immunology and Transplantation, Rega Institute for Medical Research, KU Leuven; Instituto de Medicina Tropical 'Alexander von Humboldt', Universidad Peruana Cayetano Heredia; Instituto de Medicina Tropical 'Alexander von Humboldt', Universidad Peruana Cayetano Heredia; Instituto de Medicina Tropical 'Alexander von Humboldt', Universidad Peruana Cayetano Heredia; Instituto de Medicina Tropical 'Alexander von Humboldt', Universidad Peruana Cayetano Heredia; Institute of Infectious Diseases “Emilio Ribas” / Secretaria de Saúde do Estado de São Paulo; KU Leuven; University of São Paulo Medical School - USP

**Keywords:** neuroinflammation, retrovirus, neglected disease, multi-omics, ancestry

## Abstract

HTLV-1 is an enigmatic retrovirus triggering a debilitating neuroinflammatory disease, HTLV-1-associated myelopathy (HAM), with unknown pathogenesis. Both HTLV-1 infection and HAM predominantly affect women and non-white neglected populations. HAM is lacking disease-modifying treatment, as current treatment is mostly symptomatic and inspired by either HIV-1 or multiple sclerosis therapeutic strategies. We used systems biology analyses of novel and publicly available data comprising (epi)genomics, transcriptomics, metabolomics and proteomics of multi-ancestry cohorts from a total of > 2500 People Living with HTLV-1 from 5 countries (Brazil, Peru, Japan, UK, US). Leveraging an unique admixed Brazilian cohort, genome-wide association study (GWAS) revealed African-specific variants in inflammasome sensor *AIM2* with genome-wide significance (p < 5x10^−8^). Suggestive loci (p > 5x10^−8^) corresponding to metabolic, immune and neuronal genes were validated using published Japanese GWAS. Polygenic risk score and proviral load were independent disease predictors across ancestries. Systems biology analysis revealed neuronal/synaptic signaling, monocyte count, glucose/lipid metabolism, and neurocognition/depression as genetically linked to HAM. *In silico* drug screening identified estrogen blocker Fulvestrant as the top hit, while also confirming existing (pre)clinical data for HDAC inhibitors and immunosuppressants. Validated GWAS genes were overexpressed in HAM patients’ whole blood and CD4 T-cells, as well as in spinal cord astrocytes, oligodendrocytes, and microglia by single-cell RNAseq. We experimentally confirmed decreased ApoA1/lipid/cholesterol levels, higher monocyte levels and lower neurocognitive scores in multi-ancestry cohorts. We found striking biological similarities between retroviral Hbz/Tax overexpression, Hbz interactome and HAM multi-omics findings: enrichment for lipid/cholesterol metabolism, estrogen signaling, neurodegenerative diseases, and viral pathways including EBV, recently identified as the major driver of multiple sclerosis.

In conclusion, our data-driven approach uncovers novel disease mechanisms and therapeutic targets, and a validated polygenic risk score allowing targeted surveillance for high-risk individuals. A strong molecular overlap to other neurodegenerative/neuroinflammatory diseases reveals shared neuropathogenic pathways between unrelated viruses.

Although it infects 10–20 million people worldwide, Human T-lymphotropic Virus-1 (HTLV-1) remains an enigmatic and neglected retrovirus. There is limited research, public awareness, and prevention effort^1^ and no effective treatment or vaccine. HTLV-1 prevalence increases as economic inequality increases and it infects predominantly non-Whites, females, and those with lower income and educational level^2^. HTLV-1 increases all-cause mortality^3^ and triggers high mortality and morbidity secondary diseases Adult T-cell Leukemia^4^ and HTLV-1-associated myelopathy (HAM)^5^.

HAM is characterized by progressive spastic weakness of the lower limbs, lower back pain and urinary symptoms^6^. The initial inflammatory phase is characterized by perivascular lymphocytic infiltration of the spinal cord, followed by irreversible scarring, atrophy and neurodegeneration^7^. The molecular and cellular mechanisms of HAM pathogenesis remain poorly understood, but there is strong evidence that host genetics play a significant role in its development^7^.

People Living with HTLV-1 (PLwHTLV) represent specific risk factors related to retroviral transmission and predominantly belong to neglected populations^8-10^. Therefore, for genetic studies on HAM, PLwHTLV but neurologically asymptomatic are more appropriate controls than general population controls, limiting the feasibility of assembling large, well-powered cohorts. The only published GWAS investigated > 1,500 PLwHTLV, a formidable effort for a neglected disease, and revealed genome-wide significant associations solely within the HLA class I and II regions^11^.

Replication of these findings might be complicated by epidemiological differences in disease manifestation, e.g. a younger age of onset in Latin America and a link between infective dermatitis in childhood and subsequent HAM, unique to Afro-Caribbean populations^12^. The Japanese GWAS findings underscore our current understanding of the CD4 vs. CD8 T-cells as major deleterious and protective players in HAM, representing the major retroviral reservoir and the major antiviral effector cells, respectively^11^. However, molecular and mechanistic links to the prototypical spinal cord inflammation in HAM are lacking, thus limiting novel therapeutic targets for clinical trials. In a global collaborative effort, we used a data-driven approach to gain insight into HAM pathogenesis, integrating novel and publicly available (epi)genomic, metabolomic, transcriptomic and proteomic data of several independent cohorts across ancestries.

## RESULTS

### GWAS in admixed Brazilians reveals ancestry-specific HAM loci while polygenic risk scores replicate across cohorts

**Figure 1:**
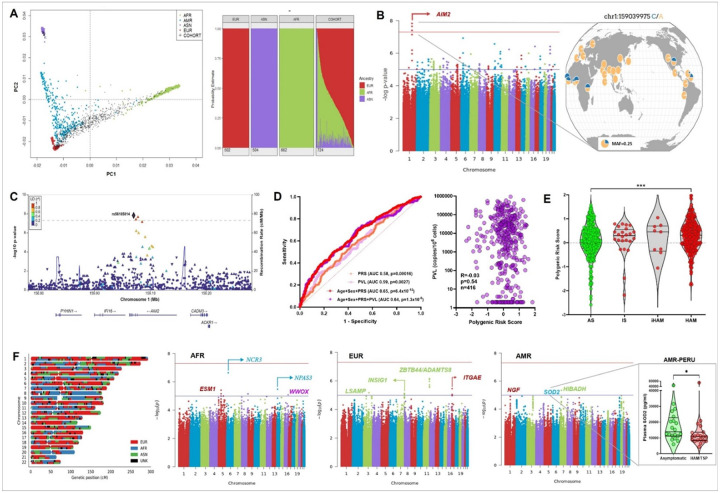
GWAS in admixed Brazilians reveals ancestry-specific HAM loci while polygenic risk scores replicate across cohorts A) The Brazilian cohort displayed mainly European (EUR) and African (AFR) admixture, with smaller contributions from Amerindian (AMR) and Asian (ASN) ancestries, as shown by PCA (left panel) and ADMIXTURE (right panel). B) Manhattan plot from Regenie GWAS showing resulting associations when using HAM status as a phenotype with logistic mixed modeling. The red horizontal line indicates the genome-wide significant threshold, and the blue horizontal line indicates the genome-wide suggestive threshold. C) LocusZoom plot of genome-wide significant variants from Regenie HAM GWAS, showing the significance of tested SNPs with HAM on the left y-axis. The right y-axis is the recombination rate. Each point represents a different SNP tested. The blue line is the level of recombination rate. The color of each point represents the strength of LD with the lead SNP. The shape of each point represents the type of SNP: splice variant (triangle), nonsynonymous (inverted triangle), synonymous (square), untranslated region (square), and none-of-the-above (circle) The lead SNP is labeled (diamond). Below are the genes at their corresponding genomic position. D) ROC curves (left panel) showing significant prediction of AS vs. HAM clinical status by univariate and multivariate logistic regression, comparing PRS and PVL, with and without age and sex correction. No correlation was observed between PRS and PVL (Spearman correlation, right panel)3. E) Violin plot overlaid with individual scores comparing the PRS distribution grouped by disease status: asymptomatics (AS), intermediate syndrome (IS), incident HAM (iHAM) and HAM (p<0.0001, AS vs. HAM, Mann-Whitney test). F) Example of TRACTOR analysis in a n a three-way admixed individual, individual stretches of each ancestry are heterogeneously dispersed throughout all chromosomes. G) Manhattan plots from TRACTOR GWAS in AFR, EUR and AMR ancestries, showing resulting associations when using HAM status as a phenotype with local ancestry-informed analysis. The red horizontal line indicates the genome-wide significant threshold, and the blue horizontal line indicates the genome-wide suggestive threshold. Right panel inset shows validation differential expression of plasma SOD2 protein in an independent Amerindian cohort of PLwHTLV (Lima, Peru, Mann-Whitney, p=0.02).

Admixed populations provide increased power for genetic discovery when ancestry is appropriately accounted for ^13^. To leverage the unique admixture of the Brazilian cohort, we used two complementary approaches: mixed modeling with Regenie and local ancestry-informed analysis with TRACTOR. The cohort displayed mainly European and African admixture, with smaller contributions from Amerindian (AMR) and Asian (ASN) ancestries, as shown by PCA and ADMIXTURE plots (Fig. 1A, left and right panel). Using the Regenie model, we identified 54 distinct loci reaching the genome-wide suggestive threshold (p-values between 3x10^−7^ and 9x10^−5^, listed in Suppl. Table 1), and one genome-wide significant (p < 5x10^−8^) association at the *AIM2* (encoding cytosolic DNA sensor and inflammasome activator Aim2) locus (Fig. 1B Manhattan plot). The lead SNP, rs56185814, associated with an increased HAM risk (OR = 2.73, 95% CI = 1.91–3.89, p = 1.49x10^−8^), is intronic and may result in differential splicing. This SNP is rare in ASN populations (1000 Genomes, inset Fig. 1B), explaining its absence in the Japanese GWAS, but has frequencies of 0.50, 0.07, and 0.05 in AFR, AMR, and EUR populations, respectively. Genome-wide suggestive and significant variants in strong LD with rs56185814 clustered between recombination hotspots (Fig. 1C). Q-Q plots confirmed adequate control of stratification (Suppl. Data).

In contrast to the Japanese GWAS, none of the identified loci were in the HLA region. Using PRSice and lassosum, polygenic risk scores (PRS) based on Japanese GWAS summary statistics were validated. PRSice PRS provided the strongest discrimination between HAM cases and asymptomatic individuals (p = 3.36x10^−5^), whereas lassosum results were significant but less pronounced (p = 0.026, data not shown). Using multivariable logistic regression and correcting for age and sex, PRS (OR 1.67, 95% CI 1.31–2.14, p = 3.9x10^−5^) and proviral load (OR 1.19, 95% CI 1.06–1.35, p = 0.0038) were found to be independent predictors of HAM disease (Suppl. Table 2). However, age + sex + PRS (AUC 0.65, p = 6.4x10^−12^) predicted HAM equally well as age + sex + PRS + PVL (AUC 0.64, p = 1.3x10–8) in ROC curve analysis (Fig. 1D). PRS was also not correlated to PVL in Brazilian PLwHTLV (R = 0.03, p = 0.54, Fig. 1D), again arguing against possible confounding. To further test its predictive power, we compared PRS between AS that remained asymptomatic upon long-term follow-up, those that present with prodromal “intermediate syndrome” (IS)^14^, incident HAM cases (iHAM)^15^ and those with established HAM (Fig. 1E). A significant difference was observed for AS vs. HAM (p < 0.001), while most IS (n = 23) and IHAM (n = 9) cases presented with a positive PRS but numbers were too small to reach statistical significance. Although women are more susceptible to HAM, median PRS between males and females were not significantly different for any PRS (data not shown). Thus, although top hits in both cohorts diverge strongly, less significant genetic variants actually replicated between Brazil and Japan when using polygenic risk score prediction.

Next, we used TRACTOR to perform 4 additional GWAS, focusing on local admixture for each of the four ancestries (AFR, EUR, AMR, ASN). As exemplified in Fig. 1G in a three-way admixed individual, individual stretches of each ancestry are heterogeneously dispersed throughout all chromosomes, e.g. chromosome 8 and 20 display long AFR stretches while the remaining chromosomes show predominantly intertwined EUR and ASN stretches. The AFR and EUR based GWAS each had two genome-wide suggestive associations related to immunological and or neurological processes. For AFR ancestry, significant associations included *NCR3* (natural cytotoxicity triggering receptor 3, OR = 0.29, p = 2.34e-7) and *NPAS3* (neuronal PAS domain protein 3, OR = 0.34, p = 3.34e-6), both protective against HAM development (Table S3). *NCR3* encodes the NKp46 activating receptor that natural killer cells use to kill target cells^16^, interacting with T-cell receptor CD247. The variant is located within the regulatory region of *NCR3,* possibly influencing the expression level. Confirming our findings, a recent study demonstrated decreased expression of NKp46/NCR3 expression by flow cytometry in Afro-descendant HAM patients from Salvador-Bahia, the largest Afro-descendant city in the Americas^17^. *NPAS3* encodes a protein domain utilized by a family of PAS transcription factors which regulate neurogenesis^16^. Abnormalities in the coding portion of this gene are known to be involved in cognitive disability, which is a possible result of HAM development. while abnormalities in the coding portion of this gene are known to be involved in cognitive disability. The lead variant is an intronic variant. For EUR ancestry, significant loci included *ITGAE* (integrin subunit α E, OR = 5.21, p = 3.50e-06) and *INSIG1* (insulin induced gene 1, OR = 2.68, p = 7.49e-06) (Table S4). *ITGAE* encodes a dimer of integrin proteins which are integral membrane proteins^16^. Integrins and corresponding T-cell ligands have been found to be involved in the transmigration of CD4 + T-cells that have been infected with HTLV-1 into CNS tissue ^18^. The lead SNP is an intronic SNP. Meanwhile, the lead SNP for *INSIG1* is located within the regulatory region of the gene. The resulting protein is associated with the endoplasmic reticulum where it regulates lipogenesis and cholesterol metabolism^16^. For AMR ancestry (Suppl. Table S5), suggestive hits included *NGF* (Nerve Growth Factor), *HIBADH* (mitochondrial 3-hydroxyisobutyrate dehydrogenase enzyme) and *SOD2* (mitochondrial superoxide dismutase2). Increased plasma NGF levels have been linked to HAM^19^. We validated differential expression of plasma SOD2 protein in an independent Amerindian cohort of PLwHTLV (Lima, Peru). As shown in Fig. 1F, we observed a 30% decrease in HAM patients compared to asymptomatic (AS) individuals (Mann-Whitney, p = 0.02), indicating a decreased systemic antioxidant capacity in HAM. As expected, due to the low percentage of ASN ancestry in our cohort, mostly representing second to third generation Japanese descendants with low admixture (clearly separated in Fig. 1A), no genome-wide suggestive SNPs/genes were identified when performing TRACTOR for ASN (Suppl. Table S6).

### Analysis of shared genes and pathways between Brazilian and Japanese GWAS reveals novel neuronal and immune links to HAM pathogenesis

**Figure 2:**
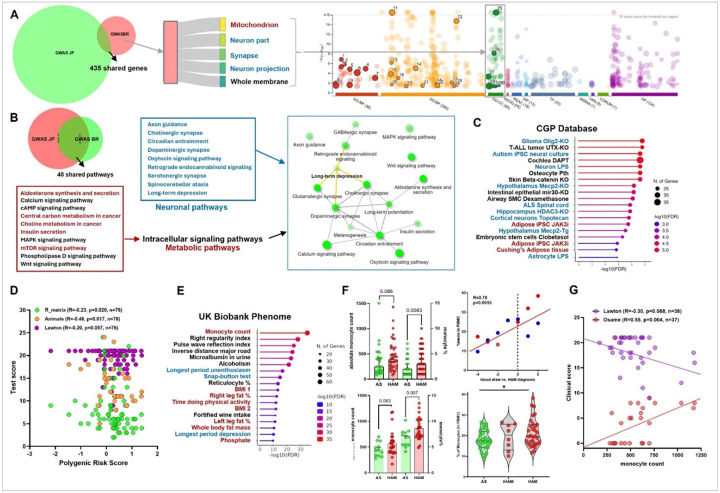
Systems biology analysis of shared genes and pathways between Brazilian and Japanese GWAS reveals novel neuronal and immune links to HAM pathogenesis A) Out of 2687 genes identified by FUMA using Regenie Brazilian GWAS output, 435 overlapped with the Japanese GWAS at nominal p-values (Venn diagram, left panel). Gene Ontology (GO) analysis of Brazilian GWAS showed a strong enrichment for mitochondria, neuron/synapse and cell membrane (middle panel). Boxes on the right represent the enriched GO biological processes. The size of each box is proportional to the total width of the connections it is a part of. The width of each connection is proportional to the −log10 p-value returned from GO enrichment utilizing the corresponding gene set from the listed GWAS. Enrichment was confirmed when examining only the 435 overlapping genes (right panel), with comprehensive enrichment analysis using publicly available databases (including gene ontology (GO), KEGG/Reactome pathways, transcription factors motifs, human phenotype ontology and drug databases). B) Venn diagram shows a strong overlap at the pathway level, with 40 shared between Brazilian and Japanese GWAS (upper panel). Pathways grouped into three major classes: intracellular signaling (black), metabolic pathways (red), and neuronal pathways (blue). Pathways were highly interconnected when plotted as a network based on overlapping genes (lower panel, boxed inset). C) Enrichment of shared GWAS genes in the CGP (Chemical and Genetic Perturbations) database confirmed and extended the observed metabolic and neuronal associations at the cellular and disease level. D) Spearman correlation analysis between HAM PRS and a complete battery of neuropsychological tests performed in a subgroup of the Brazilian cohort (n=76), significant negative correlations between HAM PRS and working/spatial memory (Animals and R_matrix tests, both p<0.05), a trend was observed for activities of daily living (Lawton test, p=0.057). E) Phenome-wide enrichment analysis in the UK Biobank identified monocyte count as the top hit, alongside associations with broad neurological phenotypes (alcohol use related traits, anhedonia and depression) and neurocognitive skills (Snap-button test). F) Validation of the genetically predicted link of HAM to blood monocyte levels in two independent cohorts (Brazil and Peru). Absolute monocyte counts were increased in HAM vs. AS in both cohorts, but the most significant increase was observed in monocyte percentage (normalized to total leukocytes, p<0.01 for both cohorts). G) Blood monocyte counts at baseline were predictive of clinical outcome during long-time follow-up, with higher monocyte levels predicting worse disease progression (Osame Motor Disability Scale, p=0.004) as well as lower activities of daily living (Lawton, p=0.068).

Using FUMA, genes from significant and suggestive loci in the Brazilian GWAS were identified based on eQTLs and chromatin structure^20^, allowing for long-range interactions since a CTCF motif is inserted into each integrated HTLV-1 proviral copy^21^. From the Regenie output, 2687 genes were identified, 435 of which overlapped with the Japanese GWAS at nominal p-values (Fig. 2A). Using Gene Ontology (GO), Brazilian GWAS showed a strong enrichment for mitochondria, neuron/synapse and cell membrane, hinting at energy metabolism and cellular signaling in the CNS, which was confirmed when examining only the 435 overlapping genes (Fig. 2A right panel). Comprehensive enrichment analysis using publicly available databases (including gene ontology (GO), KEGG/Reactome pathways, transcription factors motifs, human phenotype ontology and drug databases; see Online Methods), revealed even stronger overlap at the pathway level, with 40 shared between Brazilian and Japanese GWAS (Fig. 2B). These pathways grouped into three major classes: intracellular signaling (calcium, cAMP, MAPK mTOR, PLD and Wnt signaling), metabolic pathways (choline, insulin, central carbon metabolism, and neuronal pathways (axon guidance, cholinergic/dopaminergic/serotonergic synapse, and long-term depression). In support of common HAM disease mechanisms, these pathways were highly interconnected when plotted as a network based on overlapping genes (Fig. 2B, inset). Screening the CGP (Chemical and Genetic Perturbations) database confirmed the observed neuronal associations at the cellular (glia, neurons, astrocytes) and disease level (autism, Amyotrophic Lateral Sclerosis (ALS), Fig. 2C). In addition, CGP enrichment extended the metabolic links to Cushing’s syndrome and adipose tissue, as well as pro- and anti-inflammatory molecular links (LPS, glucocorticoids).

Phenome-wide screening in the UK Biobank (Fig. 2E) identified monocyte count as the top hit, alongside associations with broad neurological phenotypes (alcohol use related traits, anhedonia and depression) and neurocognitive skills (Snap-button test for reaction time). While the link between HAM and depression has been shown before^22-24^, a genetic neurocognitive link has not been explored yet. Thus, we examined a possible correlation between HAM PRS and a complete battery of neuropsychological tests performed in a subgroup of the Brazilian cohort (n = 76). We observed significant negative correlations between HAM PRS and working/spatial memory (Animals and R_matrix tests, both p < 0.05, Fig. 2D), while a similar trend was observed for activities of daily living (Lawton, p = 0.057). Next, we validated the link to blood monocyte levels in two independent cohorts (Brazil and Peru). Although strong inter-individual and inter-cohort differences were observed (Fig. 2F), absolute monocyte counts were increased in HAM vs. AS in both cohorts, while the most significant increase was observed in monocyte percentage (normalized to total leukocytes, p < 0.01 for both). Moreover, we found that monocyte counts at baseline were predictive of clinical outcome during long-time follow-up, with higher monocyte levels predicting worse disease progression (Osame Motor Disability Scale, p = 0.004) as well as lower activities of daily living (Lawton, p = 0.068), considering all patients for whom baseline blood counts were available.

### Validation of GWAS predictions by multi-cohort transcriptomics and immune biomarker screening

**Figure 3:**
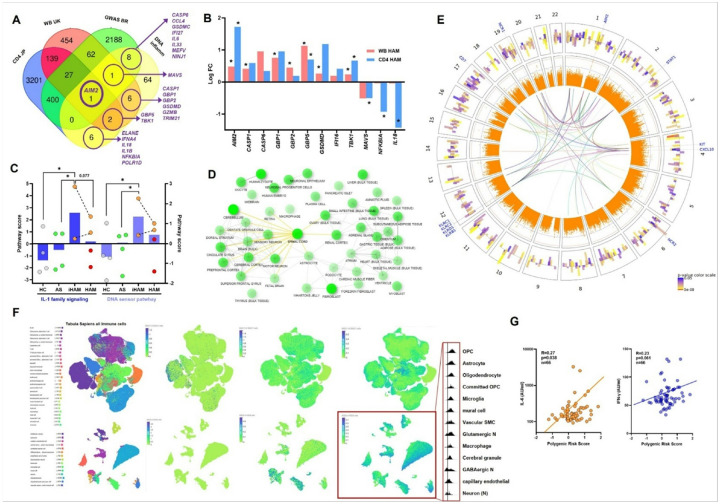
Validation of GWAS predictions by multi-cohort transcriptomics and immune biomarker screening A) Venn Diagram showing overlap between Brazilian GWAS genes identified by FUMA, transcriptomic analysis of differentially expressed genes in HAM in CD4 cells ^25^(Japanese cohort, “CD4 JP”) and in whole blood^26^ (UK cohort, “WB UK”), and KEGG-defined pathways (“DNA inflamm”: cytosolic sensing of pathogen DNA and inflammasome activation). B) From the cytosolic DNA sensing and inflammasome activation KEGG-defined genes, 15 genes overlapped with Brazilian GWAS genes, of which 8 were consistently upregulated in both “CD4 JP” and “WB UK “ data sets (all p<0.05 GEO2R analysis). C) Digital transcriptomic analysis of the US cohort ^27^ showed higher pathway scores for IL-1 signaling and cytosolic DNA sensing in 2 unique incident HAM (iHAM) cases (One-sample t-test, p<0.05 for all), as compared to uninfected healthy controls (HC) or asymptomatic PLwHTLV (AS), which remained elevated after HAM diagnosis (individual iHAM cases are connected with dotted lines before and after HAM diagnosis). D) Transcription factor "upstream” analysis of 428 “HAM CD4” genes (as defined in A) across several tissues confirmed enrichment in brain and spinal cord. E) Circos plot combining GWAS, transcriptome data and interactome data showing *AIM2, KIT, CD7 and* NK cell-related genes (KLRs, NCRs) as major hubs in the HAM protein-protein interaction network (STRING database, middle part, all FDR-corrected p<0.05). Significant protein-protein interaction with previously known HAM biomarkers *STAT1*^26,28^ and *CXCL10*^29-31^ confirms the pathobiological significance of GWAS findings. F) Tabula Sapiens whole-body single-cell atlas (CellxGene) was used to map HAM genes prioritized by both GWAS and transcriptomics. Among immune cells (upper panels), we found sparse expression of *AIM2* in B-cells > neutrophil > plasma cells >activated T-cells, while the “HAM inflammasome” gene module (as defined in B) was ubiquitously expressed with Tregs>macrophages>monocytes>NK cells. Exploring a spinal cord single-cell dataset (CellXgene), we found no detectable AIM2 but the “HAM inflammasome” gene module (as defined in B) was modestly expressed in microglial cells>endothelial cells>macrophages>oligodendrocyte precursors>astrocytes (lower panels). Exploring the “broad HAM” gene module (95 genes shared between Brazilian and Japanese GWAS, all overexpressed in HAM CD4 cells), we observed modest expression among whole-body immune cells (upper right panel: highest in macrophages>monocytes>microglial cells>B-cells), but strong and widespread expression across all cell types in the spinal cord (lower right panel: oligodendrocyte precursor cells (OPC) >astrocytes >oligodendrocytes >microglia >mural cells> vascular smooth muscle cells> glutamergic neurons> macrophages> cerebral granule cells>GABAergic neurons >capillary endothelial cells >neurons). Inset (lower right panel) shows the quantification of all 95 genes in spinal cord cell types as density plots. G) Spearman correlation analysis of HAM PRS and circulating plasma cytokine levels for IL-6 (p=0.038) and plasma IFN-γ (p=0.061) in 66 patients for which paired samples were available.

To further validate our GWAS findings, we tested unique as well as shared genes between Japanese and Brazilian cohorts for differential expression in three independent transcriptomic data sets: HAM CD4 cells (the main HTLV-1 reservoir) from Japanese HAM patients ^25^, HAM whole blood from an Afro-Caribbean UK cohort^26^, and our own samples from the US HOST cohort^27^ (with >15 years follow-up). For the latter, we had access to unique incident HAM (iHAM) cases with paired samples before and after diagnosis. As shown in Fig. 3A (left panel), a total of 428 GWAS genes were validated as differentially expressed in HAM CD4 + cells, and 91 in HAM whole blood (Suppl. Table S7), of which 28 genes were shared. These shared genes included *AIM2,* hence validating our GWAS top hit in two independent data sets from different ancestries. Therefore, we examined possible overexpression of other genes with AIM2-related biological function, i.e. cytosolic DNA sensing and inflammasome activation (88 genes defined by KEGG), resulting in 15 additional overlapping genes, of which 8 were consistently upregulated in both data sets: canonical activator proteases caspase-1 (*CASP1*) and gasdermin D (*GSDMD*) as well as *CASP6, GBP1, GBP2, GBP5, IFI16* and *TBK1* (Fig. 3B). Digital transcriptomic analysis of the US cohort allowed pathway score quantification of IL-1 signaling and cytosolic DNA sensing, which were both significantly higher in iHAM cases, as compared to uninfected healthy controls (HC) or asymptomatic PLwHTLV (AS), and remained elevated after HAM diagnosis (Fig. 3C).

Enrichment analysis of 428 “HAM CD4” (as defined in Fig. 3A) genes showed a predominance of neuronal pathways and gene networks, revealing unexpected expression of presumed neuronal genes in CD4 + T-cells. To approach this mechanistically, we investigated upstream transcription factors (TFs) to these 428 genes across several tissues, which confirmed enrichment in brain and spinal cord (Fig. 3D), the major target of HAM inflammation and tissue damage. As can be observed from the Circos plot combining GWAS, transcriptome data and interactome data (Fig. 3E), *AIM2, KIT, CD7 and NK* cell-related genes (KLRs, NCRs) are major hubs in the HAM protein-protein interaction network (STRING database, middle part of Fig. 3E). In addition, they significantly interact with previously known HAM biomarkers such as *STAT1* ^26,28^ and *CXCL10*^29-31^, underscoring the pathobiological significance of our GWAS findings.

Next, we explored the Tabula Sapiens whole-body single-cell atlas to map HAM genes prioritized by both GWAS and transcriptomics. Among immune cells (Fig. 3F upper panels), we found sparse expression of *AIM2* in B-cells > neutrophil > plasma cells > activated T-cells, while the HAM inflammasome gene module (as defined in Fig. 3B) was ubiquitously expressed with Tregs > macrophages > monocytes > NK cells. Although differences were subtle, both *AIM2* and the inflammasome module showed higher expression in females (data not shown) and displayed minor ancestry differences (ASN > AMR > AFR > EUR for *AIM2,* AFR > EUR > ASN > AMR for the “HAM inflammasome” gene module, data not shown). Exploring a spinal cord single-cell dataset (CellXgene), we found no detectable *AIM2* but the “HAM inflammasome” gene module (as defined in Fig. 3B) was modestly expressed in microglial cells > endothelial cells > macrophages > oligodendrocyte precursors > astrocytes (Fig. 3F lower panels). Lastly, exploring the “broad HAM” gene module (95 genes shared between Brazilian and Japanese GWAS, all overexpressed in HAM CD4 cells), we observed modest expression among whole-body immune cells (highest in macrophages > monocytes > microglial cells > B-cells), but strong and widespread expression across all cell types in the spinal cord (oligodendrocyte precursor cells (OPC) > astrocytes > oligodendrocytes > microglia > mural cells > vascular smooth muscle cells > glutamergic neurons > macrophages > cerebral granule cells > GABAergic neurons > capillary endothelial cells > neurons), again underscoring its putative role in neuroinflammation. In keeping with HAM risk factors, expression of the “broad HAM” gene module in spinal cord was higher in female vs. male and old vs. young individuals (data not shown).

To explore upstream cytokines regulating the HAM gene signature, we examined possible correlation between PRS and a total of 43 immune mediators ^32^. We found plasma IL-6 was significantly correlated (p = 0.038, Fig. 3G) to PRS, confirming Regenie GWAS findings, while a trend was observed for IFN-γ (p = 0.061).

Lastly, *in silico* drug screening of HAM GWAS genes identified FDA-approved estrogen blocker Fulvestrant as the top hit, in addition to progesterone (Suppl. Figure 3), providing a molecular mechanism for the increased female HAM susceptibility. In addition, *in silico* identification of HDAC inhibitors entinostat, vorinostat and panabinostat confirm promising preclinical findings on the use of this drug class to treat HTLV-1 infection and possibly HAM patients^36^. Of interest, the older HDAC inhibitor valproate, which is safe but ineffective in HAM patients^37^ was not significantly enriched in our analysis.

### Genetic links to lipid metabolism and proviral load are validated by in vivo metabolomics and in vitro retroviral Tax/Hbz overexpression and interactome

**Figure 4:**
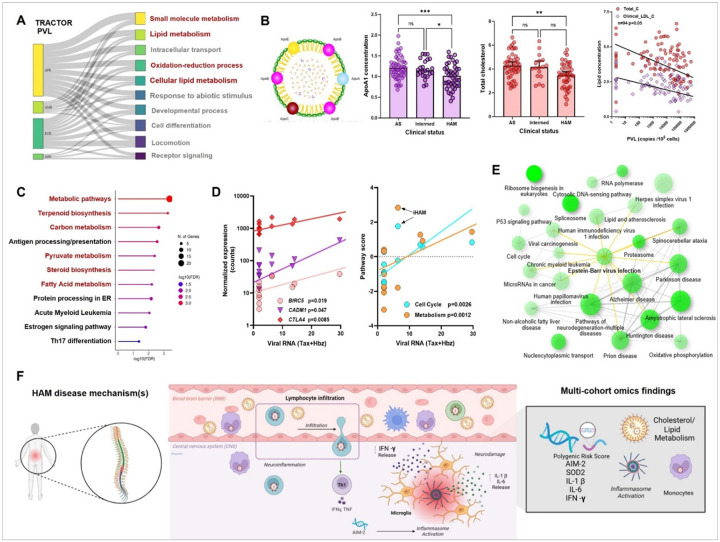
Genetic links to lipid metabolism and proviral load are validated by *in vivo* metabolomics and *in vitro* retroviral Tax/Hbz overexpression and interactome A) Using log-transformed PVL as a phenotype, 841, 27, 11, 402, and 162 GO terms were enriched for AFR, AMR, ASN, EUR, and Regenie datasets, respectively. AFR and EUR PVL GWAS showed the strongest enrichment, highlighting small molecule and lipid metabolism as well as oxidation-reduction processes. B) ^1^H-NMR metabolomic analysis in a subset of the Brazilian GWAS cohort (n=110) showed lower levels of lipid metabolites in HAM compared to AS, including ApoA1 (p<0.001), ApoB (p<0.05), total (p<0.01) and HDL-cholesterol (p<0.05). ApoA1 levels were already decreased in patients with prodromal intermediate syndrome (Intermed, *p<0.05), suggesting an early event in HAM pathogenesis, Consistent with PVL GWAS enrichment analysis, proviral load was inversely correlated with total and clinical LDL-cholesterol levels (p<0.05 for both). C) Pathway enrichment analysis of all genes upregulated by both Tax and Hbz overexpression in Jurkat cells. D) Validation of a shared molecular imprint of viral Tax and Hbz using digital transcriptomic analysis in the US cohort. Total viral RNA (Tax+Hbz) levels were positively correlated to cell cycle (p=0.0012) and metabolism pathway scores (p=0.0026), as well as several transcripts corresponding to GWAS genes (*CTLA4, CADM1, BIRC5*, al p<0.05). E) Enrichment analysis of the HBZ human interactome revealed striking biological similarities to HAM GWAS findings: cell cycle, lipid metabolism and oxidative phosphorylation pathways were confirmed, as well as several viruses that can cause neurodegeneration (HIV-1), encephalitis (HSV-1), and Epstein-Barr virus (EBV), recently identified as the major driver of neuroinflammation in multiple sclerosis^34^. EBV infection was the most central pathway (yellow nodes) in the network, with strong molecular overlap to other neurodegenerative diseases (spinocerebellar ataxia, ALS, Alzheimer, Parkinson and prion disease). F) Proposed HAM disease mechanism(s) of spinal cord infiltration and inflammation triggered by the combined effects of AIM2 inflammasome activation, monocyte and microglial activation, proinflammatory cytokine secretion (IL1γ, IL-6, TNF, IFN-γ), accompanied by decreased antioxidant capacity mitochondrial (SOD2) and reprogramming of (systemic and/or local) lipid metabolism.

In contrast to the HAM GWAS, only 14 loci reached suggestive significant in the log-transformed PVL-based GWAS using Regenie (Suppl Table S8). Using log-transformed PVL as a phenotype, 841, 27, 11, 402, and 162 GO terms were enriched for AFR, AMR, ASN, EUR, and Regenie datasets, respectively (Fig. 4A). Unlike HAM, AFR and EUR PVL GWAS showed the strongest enrichment, highlighting small molecule and lipid metabolism as well as oxidation-reduction processes. Among these, *APOB,* encoding one of the major components of circulating chylomicrons (Fig. 4B), was genome-wide suggestive in both Regenie and EUR PVL GWAS. Therefore, we performed ^1^H-NMR metabolomic analysis in: a subset of the Brazilian GWAS cohort (n = 110). Surprisingly, we found lower levels of major lipid metabolites in HAM compared to AS, including ApoA1 (p<0.001), ApoB (p<0.05), total (p<0.01) and HDL-cholesterol (p< 0.05) (Fig. 3B), sphingomyelins and phospholipids (Suppl. Table S9). In addition, glycolytic metabolites and amino acids (Suppl. Table S9) were strongly perturbated in HAM. Consistent with PVL GWAS enrichment, proviral load was inversely correlated with total and clinical LDL-cholesterol levels (p < 0.05 for both, Fig. 4B).

To test the applicability of trans-ancestry PRS for metabolic phenotypes, we utilized UK Biobank GWAS data (using the same ^1^H-NMR platform, Nightingale Health) to generate PRS for acetate and ApoA-I. The optimized p-value threshold when using lipid levels as a phenotype are detailed in Online Methods. PRS for acetate and ApoA-I showed significant correlations with their respective metabolite levels (Pearson’s R = 0.28, p = 0.016 for both, Suppl. Figure 3A-B).

Proviral load (PVL) is a measure of the total integrated HTLV-1 in the host genome, which remains mostly inactive but transcription can occur in sporadic bursts^33^ Although the exact molecular mechanisms are largely unknown, both *Tax* and *Hbz* viral oncogenes are presumed to play a major role in HAM pathogenesis. Thus, we examined the *in vivo* genetic and transcriptomic HAM signatures for a possible overlap with the human Tax and Hbz protein interactome, as well as genes regulated in vitro by overexpression of Tax and Hbz in Jurkat cells. Among all genes upregulated by both Tax and Hbz overexpression, we found significant enrichment of several pathways shared with our HAM findings (Fig. 4C): metabolic pathways (pyruvate, steroids, fatty acids) as well as immune pathways (antigen presentation, Th17 activation) and estrogen signaling (in agreement with female predominance in HAM). Thus, a large part of our multi-omic findings appear to carry a shared molecular imprint of viral Tax and Hbz. We validated this finding using digital transcriptomics, allowing sensitive detection of Tax and Hbz RNA. As shown in Fig. 4D, total viral RNA (Tax + Hbz) levels were positively correlated to cell cycle (p = 0.0012) and metabolism pathway scores (p = 0.0026), as well as several transcripts corresponding to GWAS genes (*CTLA4, CADM1, BIRC5,* al p< 0.05). In addition, enrichment analysis of the Hbz vs. Tax human interactomes revealed striking biological similarities between the Hbz interactome and HAM GWAS findings. Cell cycle, lipid metabolism and oxidative phosphorylation pathways were confirmed, as well as several viruses that can cause neurodegeneration (HIV-1), encephalitis (HSV-1), and Epstein-Barr virus (EBV), recently identified as the major driver of neuroinflammation in multiple sclerosis^34^.

Moreover, EBV infection was the most central pathway in the network, with strong molecular overlap to other neurodegenerative diseases (spinocerebellar ataxia, ALS, Alzheimer, Parkinson and prion disease), hinting at shared neuropathogenic pathways between unrelated viruses.

## DISCUSSION

HTLV-1-associated myelopathy remains a neglected neuroinflammatory disease, with a poorly understood pathogenesis and no disease-modifying treatments. Through a global, multi-omic analysis, this study provides novel insights into the genetic, immunological, and metabolic underpinnings of HAM, leveraging genomic and transcriptomic datasets across ancestries. This represents the largest integrative molecular study of HAM, underscoring the importance of including underrepresented populations in genomic research.

Using ancestry-aware GWAS in a unique admixed Brazilian cohort, we identified *AIM2* as a genome-wide significant locus, a result not replicated in the Japanese GWAS due to the rarity of the genetic variant rs56185814 in Asian cohorts. *AIM2* encodes a cytosolic DNA sensor central to inflammasome activation and IL-1 p maturation, implicating innate immunity in HAM pathogenesis. This finding aligns with the prototypical spinal cord inflammation characteristic of HAM and highlights ancestry-specific genetic mechanisms of disease susceptibility. In contrast, the previously published Japanese GWAS reported HLA class I and II associations but lacked significant non-HLA findings, emphasizing the need for ancestry-adjusted analyses and multi-ethnic validation^11^.

Beyond *AIM2,* our systems biology approach revealed suggestive loci linked to innate immunity mediated by monocytes and NK cells (KLRs and *NCR3*), upstream cytokines IL-6 and IFN-γ, neuronal pathways (*AUTS2, NGF, NPAS3*), and lipid metabolism (*LDLR, CD36, HMGCR, LPIN2*). The latter was experimentally validated in metabolomic analyses showing altered lipid profiles in HAM patients, including decreased total cholesterol and HDL levels. This aligns with metabolic shifts observed in other neuroinflammatory conditions, further supporting a systemic component to HAM pathogenesis^45^.

Gene-pathway analysis across Brazilian and Japanese cohorts revealed shared enrichment in neuronal, intracellular signaling, and metabolic pathways, including axon guidance and Wnt signaling. These pathways are critical for neuroimmune interactions and align with established neurodegenerative disease mechanisms.

Of relevance to the HTLV field, our data conform and extend published findings on HAM pathogenesis at the molecular (IL6, CTCF, KDR-VEGF, TGFBR2), cellular (NK cells, monocytes)^46-49,46-49^ and metabolic (lipids and chylomicrons) level. Several groups worldwide have previously demonstrated a significant link between HTLV-1 infection, HAM and depression ^22^ which was mostly interpreted as an indirect effect of lower QoL due to the debilitating disease process^50^. However, our data suggest depression can be explained, at least partly, by a direct genetic link with HAM, emphasizing the potential for early therapeutic intervention with antidepressants.

Although more multi-omics studies of large cohorts are needed to further dissect HAM pathogenesis, our study represents an essential first step for a Precision Medicine approach for both PLwHTLV and the clinicians treating them. First, a validated polygenic risk score will help identify those at increased risk of developing HAM for a targeted clinical follow-up. Second, we identified novel disease biomarkers as well as therapeutic targets for known and novel drug candidates, both ready for fast repurposing in direly needed clinical trials for HAM patients.

Of note, PRS did not differ between the sexes, strongly implying that the increased female susceptibility to HAM is driven by hormone levels rather than genetics. This hypothesis is supported by three independent findings. First, *in silico* drug screening identified FDA-approved estrogen blocker Fulvestrant as the top hit, pointing at immediate clinical translation. Second, transcription factor enrichment identified ESR1 as upstream regulator of both the HAM genomic and transcriptomic signature. Third, in vitro cell lines treated with estradiol show significant overlap with HAM GWAS and transcriptome findings.

Despite these advances, limitations of the study should be acknowledged. Given that HAM is a neglected disease the greatest limitation is the lack of well-powered studies. This lack of power was further aggravated when using log-transformed PVL, lipid levels or neuropsychological assessment as a phenotype since far fewer samples had those measurements. To increase our potential for discovery, we opted to maximize power by not correcting HAM GWAS for PVL, but rather use PVL as a separate phenotype, as well as correcting for PVL in post-hoc multivariable logistic regression. In support of this approach, PVL GWAS indeed provided distinct biological enrichment, such as lipid metabolism. Significantly larger future GWAS studies can address this bias by using mediation analysis or Mendelian randomization to determine causality. Although this was a global collaborative effort integrating omics data from several cohorts with different ancestries, none of the cohorts had multi-omics data (e.g. combined GWAS and transcriptomics). Therefore, the major challenge of this study was validating biological findings using a cross-omics systems biology approach, focusing on biological pathways and processes rather than single genes, proteins or metabolites. HAM can still develop in some of the asymptomatic individuals, which could be introducing a bias. However, we have sampled HTLV-1-infected individuals at a median age (57 years) before which HAM usually manifests in Brazil (earlier than Japan), and have included several incident HAM cases that arose during the years between sampling and GWAS analysis^32^. Furthermore, the cross-sectional design limits causal inference, though longitudinal data from incident HAM cases in Brazilian and US cohorts partially address this gap.

Future studies should aim for larger multi-omics datasets with longitudinal follow-up to validate and expand these findings. In conclusion, our results confirm the importance to invest in genomic and multi-omics analysis of under-represented populations and their neglected diseases.

## Data Availability

All anonymized data are available from the authors upon reasonable request. GWAS summary statistics are available at https://doi.org/10.48804/ITSA6K. Custom code used in this study is available at https://github.com/isaacracine/ham_tsp_multi_omics.git.
